# Evolving Aspects of Prognostic Factors for Pediatric Cancer

**DOI:** 10.3390/diagnostics13233515

**Published:** 2023-11-23

**Authors:** Maria Kourti, Emmanouel Hatzipantelis

**Affiliations:** 1 Third Department of Pediatrics, School of Medicine, Aristotle University and Hippokration General Hospital, 54642 Thessaloniki, Greece; 2 Children & Adolescent Hematology-Oncology Unit, Second Department of Pediatrics, School of Medicine, Aristotle University of Thessaloniki, 54124 Thessaloniki, Greece; hatzip@auth.gr

Advances in risk-directed therapy based on prognostic factors that include clinical, biologic, and genetic features of cancer in children have yielded improved and prolonged responses. This Special Issue of *Diagnostics* consists of ten articles which illuminate different aspects of advances in childhood and adolescent hematology oncology, including original articles and narrative and systematic reviews.

The landmark article of this issue by Kourti et al. provides an extended review of the unanswered challenge of modern οmics technology, especially proteomics, in childhood acute lymphoblastic leukemia (ALL) [1]. Proteomic evaluation has impacted the study of solid tumors by increasing the understanding of the underlying tumor biology and, thus, developing promising therapies in the field of oncology by identifying relevant signatures for different cancers [2]. The proteomic profiling of hematologic malignancies creates the opportunity to explore gaps in disease relapse and resistance, as well as to encourage the discovery of novel biomarkers. The identification of proteins and pathways related to the environment and cancer provides an insight into tumor development with the potential to provide new prospects for precision medicine in childhood oncology. Therefore, proteomics may serve as a useful tool for creating innovative and individualized therapy by overcoming increased toxicity from the intensification of treatment in relapsed/refractory childhood ALL. 

The genetic landscape of childhood ALL has been broadly studied, yielding novel prognostic markers for risk stratification. Genome-wide technologies and the identification of gene copy-number alterations (CNAs) implicated in leukemogenesis have led to the relentless decoding of the underlying biology of pediatric ALL [3]. One of the most frequent genes affected is the CDKN2A/2B gene, serving as a secondary cooperating event and contributing to cell-cycle regulation chemosensitivity [4]. In the original study by Ampatzidou et al., the CDKN2A/2B deletion was identified as an additive independent prognostic factor for survival in children treated with contemporary BFM-based protocols that can further genetically refine risk stratification based on minimal residual disease [5]. 

The review by Ntenti et al. summarizes evidence about clinical, histopathological, and molecular factors that have an impact on the prognosis of childhood medulloblastomas (MBs), which are the most common and highly aggressive neoplasms of the central nervous system [6]. New molecular techniques, genomic and transcriptomic analyses, have played an important role in forming novel molecular subgroups of MBs outlined in the recent 2021 WHO molecular classification [7]. Nevertheless, the great heterogeneity within subgroups is challenging. As molecular and genetic pathways in the pathogenesis of MBs are further elucidated, a new risk stratification system will evolve. This landmarks the dawn of a new era in molecular-based MB stratification and prognosis with the optimal goal of developing patient-tailored therapeutic strategies.

Papillary thyroid cancer (PTC) is the most frequent histopathological type in children, with distinct characteristics and prognosis compared to that in adults [8]. This may be attributed to a discrete molecular basis in the activation of the MAPK pathway and its components. Mutated proto-oncogene B-Raf (BRAF) is the most common genetic alteration in adult PTC, and its prognostic significance has been extensively explored in adults [9]. Kotanidou et al. conducted the first systematic review and meta-analysis on the prognostic significance of BRAF gene mutational status in children and adolescents [10]. Since immunomodulation with BRAF inhibitors and molecularly targeted therapy is an appealing contemporary approach, conclusions made from this systematic review may serve as useful tools to establish guidelines in children and adolescent patients with PTC.

Craniopharyngiomas (CPs) are classified as non-malignant neoplasms but their location, growth pattern, and recurrence rate are associated with significant morbidity and mortality. The aim of the extensive narrative review by Serbis et al. is to identify clinical, morphological, and immunohistochemical factors that are associated with the onset and, mainly, the recurrence of CP [11]. Molecular features such as BRAF gene mutations, altered p53 expression, increased Ki-67 expression, higher VEGF and HIF1a expression, and RARs have been extensively studied as potential predictive factors for CP relapse [11]. Moreover, the decision of whether the surgical removal is followed by radiotherapy, age, adherence to surrounding tissues, histology, specific clinical findings, and molecular features remains to be validated through well-designed multicenter prospective studies, with the ultimate goal of developing targeted adjunct therapies [11].

Ovarian cancer in adolescents is challenging not only because of the rarity of the disease but also because of the particularities and substantial differences in incidence, histology, diagnostic work-up, and therapeutic management between adults and the pediatric/adolescent population. Siarkou et al., in their comparative/narrative review, made a great and valuable effort to combine and concisely summarize the existing guidelines from ESMO 2018, ESGO-SIOPE 2020, EXPeRT/PARTNER 2021, and aTRMG 2022 about the diagnosis, prognosis, and management of ovarian malignancies [12]. The reported differences highlight the need for the adoption of an international consensus to further improve the management of adolescent ovarian cancer.

Botryoid rhabdomyosarcoma (RMS), an aggressive subtype of embryonal RMS, is a rare type of tumor mainly affecting very young girls during infancy and early childhood. The narrative review by Siarkou et al. is the first to provide a comprehensive summary of the main outcomes regarding the optimal therapeutic management and prognosis of sarcoma botryoides in female children [13]. It may serve as the initial step towards the globalization of the standards of practice through prospective observational cohorts with the ultimate goal to improve outcomes of this rare but demanding clinical entity. 

The impressive increase in survival exceeding 80% for all cancer types and 90% for ALL in many European and North American countries may be attributed not only to the novel multimodal therapies but also to supportive healthcare [14]. Interesting results were achieved in a recent report which showed that mortality among cancer patients is higher than that of the general population, mainly due to increased cardiotoxicity and the development of second neoplasms [15]. Two articles in this Special Issue focus on the risk of cardiotoxicity. The original article by Ardelean et al. explores whether novel echocardiographic measures like speckle-tracking echocardiography (STE), global longitudinal strain (GLS), and the myocardial performance index (MPI) may predict early changes in cardiac function not detected through traditional methods [16]. The promising results that these novel echocardiographic measures contribute to the early detection and long-term prediction of anthracycline-induced cardiotoxicity need to be validated with further longitudinal studies. 

The early and preventive diagnosis of cardiotoxicity after chemotherapy treatment in children with cancer using omics technology is extensively reviewed by Antoniadi et al. Conventional biomarkers used for early detection were incorporated into routine diagnosis and monitoring [17]. Their main limitation was that increased levels were detected after the occurrence of significant cardiac damage. On the contrary, omics including genomics, transcriptomics, proteomics, and metabolomics offer new opportunities for biomarker discovery, providing an understanding of cardiotoxicity beyond traditional technologies. Cardio-specific miRNAs circulating in plasma are promising biomarkers for the detection of subclinical cardiotoxicity, and metabolomics have the potential to revolutionize the ability of individualized cardio-profiles shedding light on the underlying biological processes of cardiotoxicity [17]. 

Despite the established prognostic factors in childhood malignancies, nutritional status is identified as a crucial factor for optimal clinical outcomes in children with cancer, hence providing a significant modifiable prognostic tool in childhood cancer management. However, scarce studies have examined the impact of nutritional status on the survival of children with cancer, with the majority of them focusing on hematological malignancies [18]. Karalexi et al. summarized published evidence evaluating the association of under-nutrition and over-nutrition with prognosis and treatment-related toxicities (TRT) in children and adolescents treated for cancer [19]. Interestingly, the risk of death and relapse increased by 30–50% in children with leukemia and higher body mass index at diagnosis [19]. Similarly, the risk of TRT was higher in malnourished children with Ewing sarcoma and osteosarcoma. Longitudinal studies utilizing new technologies and assessing the nutritional status in a standardized way are needed in the direction of personalized interventions [19].

In conclusion, in this Special Issue, recent advances are discussed and novel prognostic markers are critically appraised. A greater understanding of the heterogeneity of pediatric cancers will ultimately lead to new therapeutic strategies with the potential to provide new prospects for precision medicine in pediatric oncology.
